# Non-inferiority comparison of medical glue and hook-wire for preoperative localization of multiple pulmonary nodules

**DOI:** 10.3389/fsurg.2025.1575698

**Published:** 2025-04-11

**Authors:** Zexin Xie, Ran Ma, Tao Gao, Jingsong Liu, Kewei Zhang, Jun Li, Xiaotong Zhou, Hao Zhang

**Affiliations:** ^1^ Thoracic Surgery Laboratory, Xuzhou Medical University, Xuzhou, Jiangsu, China; ^2^ Department of Thoracic Surgery, Affiliated Hospital of Xuzhou Medical University, Xuzhou, Jiangsu, China

**Keywords:** hook-wire, medical glue, computed tomography-guided puncture localization, multiple pulmonary nodules, video-assisted thoracoscopic surgery

## Abstract

**Background:**

Video-assisted thoracoscopic surgery is a widely employed minimally invasive approach for resecting pulmonary nodules. However, precisely localizing deep-seated or poorly visualized nodules remains a prominent challenge. This study aims to compare the effectiveness and safety of medical glue vs. hook-wire in the CT-guided localization of multiple pulmonary nodules.

**Methods:**

A prospective cohort of patients scheduled for CT-guided localization of multiple pulmonary nodules between February 2024 and August 2024 was enrolled. Patients were randomized into a medical glue group and a hook-wire group in a 1:1 ratio. The primary endpoint was the localization success rate, with the objective of conducting a non-inferiority comparison of medical glue and hook-wire for the preoperative localization of multiple pulmonary nodules at a non-inferiority margin of 10%. Secondary endpoints consisted of complication incidence following localization and pain scores assessed via the Numerical Rating Scale.

**Results:**

Medical glue demonstrated non-inferior success rates vs. hook-wire [97.5% vs. 95%, absolute difference = 2.5%, 95% CI (−5.82%, 10.82%); *P* = 1.00]. Although overall complications were comparable (42.5% vs. 55.0%, *P* = 0.263), medical glue significantly reduced pneumothorax (22.5% vs. 47.5%, *P* = 0.019) and pulmonary hemorrhage (7.5% vs. 27.5%, *P* = 0.019). Patients receiving medical glue reported lower 2 h pain scores (2.70 ± 0.76 vs. 3.71 ± 1.42, *P* = 0.007).

**Conclusions:**

The application of medical glue for the CT-guided localization of multiple pulmonary nodules demonstrated a non-inferior success rate comparable to that of hook-wire. Furthermore, compared to hook-wire, medical glue was associated with a lower incidence of pneumothorax and pulmonary hemorrhage, as well as reduced pain scores at 2 h post-localization.

**Clinical Trial Registration:**

https://www.chictr.org.cn, identifier (ChiCTR2400080040).

## 1 Introduction

The continuous improvement of socioeconomic standards has led to a growing awareness of health issues among the general population. Moreover, the increasingly widespread application of low-dose computed tomography (CT) for lung cancer screening in high-risk populations has resulted in a higher likelihood of detecting pulmonary nodules (1). CT-based lung cancer screenings have also revealed the involvement of two or more nodules in 51.5% of the participants (2). Consequently, patients presenting with multiple pulmonary nodules and requiring thoracic surgical intervention have become more frequent (3). Video-assisted thoracoscopic surgery (VATS) has been extensively adopted as the standard thoracic surgery approach for managing pulmonary nodules in clinical practice, leading to less trauma than traditional open thoracotomy (4). A successful thoracoscopic surgery critically relies on the accurate localization of the nodules. However, precise localization becomes more challenging for nodules that are deep and not directly observable or palpable near the pleura. Previous studies have reported that intraoperative difficulties in palpating or accurately locating the nodules can result in incomplete nodule excision or conversion to open thoracotomy among multiple patients (5). Nevertheless, the CT-guided localization of pulmonary nodules has significantly enhanced the success rate of thoracic surgeons performing VATS and facilitated the rapid and accurate identification of target nodules during surgery (6).

The localization of multiple nodules can present greater challenges than solitary nodules and lead to a higher complication incidence (7). Selecting appropriate materials for localizing multiple pulmonary nodules is crucial for successful localization and minimizing complication occurrence (8). Clinically, the CT-guided localization of pulmonary nodules has been performed utilizing various materials, commonly including coils, hook-wires, methylene blue, medical glue, and radioactive markers (7). Among these, hook-wire is the most broadly employed and established localization material because of its technical maturity and high success rate (6). However, recent studies have demonstrated that medical glue, due to its tissue-adhesive properties, exhibits reduced invasiveness when applied in pulmonary nodule localization, as evidenced by lower rates of pneumothorax and pulmonary hemorrhage (7, 9, 10). Hence, this study aims to compare the effectiveness and safety of medical glue vs. hook-wire in the CT-guided localization of multiple pulmonary nodules by performing a prospective non-inferiority randomized controlled trial.

## 2 Methods

### 2.1 Patients

This prospective study collected data from patients who were scheduled to undergo CT-guided localization of multiple pulmonary nodules before VATS at the Thoracic and Cardiovascular Surgery department of our hospital between February 2024 and August 2024. A total of 97 patients were initially enrolled, among which 17 were excluded because they did not meet the inclusion criteria. Ultimately, 80 patients with 178 pulmonary nodules were analyzed. Using a random number table, the patients were divided into a medical glue group and a hook-wire group in a 1:1 ratio. Inclusion criteria: patients who (1) were scheduled for CT-guided localization of nodules before thoracoscopic lung resection; (2) had ≥2 nodules in the lungs required localization evaluation by the chief physician; (3) had suspected malignant nodules with diameters <3 cm; and (4) a distance of ≤3 cm between the nodules and adjacent pleura. Exclusion criteria: patients who had (1) puncture site infection; (2) allergies to local anesthetics; (3) neuromuscular disorders or mental illness; (4) large blood vessels or bronchi adjacent to the puncture path that prevented safe localization; or (5) severe emphysema, interstitial lung disease, severe comorbidities, or contraindications for thoracoscopic surgery.

The determination of preoperative localization is made by the chief physician based on the planned surgical approach. In managing multiple pulmonary nodules, our institution follows surgical principles derived from pivotal clinical trials including JCOG0802 and CALGB140503. This approach emphasizes complete resection of the primary lesion while systematically addressing secondary nodules to achieve thorough oncological control. Lobectomy becomes the preferred option when the anatomical distribution of multiple nodules would leave inadequate functional lung tissue after resection, potentially resulting in postoperative complications such as atelectasis.

For cases where anatomical resections including segmentectomy or lobectomy are planned, preoperative localization is routinely omitted since these *en bloc* resection techniques inherently ensure complete nodule removal. The current study specifically excluded such cases, focusing instead on nodules located within 3 cm of the pleural surface that are amenable to preoperative localization.

This study was approved by the Ethics Committee of the Affiliated Hospital of Xuzhou Medical University (XYFY2023-KL446-01). Written informed consent was obtained from all included patients. The trial has been registered with the Chinese Clinical Trial Registry (ChiCTR2400080040) at https://www.chictr.org.cn.

### 2.2 Observation indicators

This study evaluated various patient characteristics, including gender, age, BMI, preoperative comorbidities, number of CT scans, procedure duration, body position, nodule location, distance from the nodule to the pleura, distance from the localization material to the nodule, nodule size, and nodule length. Additionally, data on the type of procedure, localization complications, interval duration between localization operations, surgical type, operation time, pathology, and postoperative hospitalization days were obtained. The primary endpoint of the study was the rate of successful localization, which was defined as the achievement of a distance of <2 cm between the localization material and the target nodule and no displacement of the localization material during intraoperative exploration. The secondary endpoint involved the incidence of localization-related complications, including pneumothorax, pulmonary hemorrhage, and pain scores measured at 15 min and 2 h after the localization procedure. Two independent raters assessed pain scores using a Numerical Rating Scale, where 0 points indicated no pain; 1–3 points, mild pain; 4–6 points, moderate pain; and 7–10 points, severe pain.

### 2.3 Preoperative localization

All localization procedures were conducted on the day of surgery by a single thoracic surgeon in the radiology department. The patient's position was selected based on their preoperative imaging data, followed by determining the puncture point and path. The puncture site was first disinfected, followed by local anesthesia using 5% lidocaine. The insertion direction of the trocars was set as perpendicular as possible to the skin, with all trocars adjusted to gradually penetrate 5–10 mm from the nodule edge and approximately 5 mm deep from the visceral pleura. In the medical glue group, 0.15 ml of medical glue (n-butyl *α*-cyanoacrylate) was sequentially injected into a 22G trocar, which was removed following the injection. In the hook-wire group, the trocar was sequentially moved to the distal end, extending 1 cm to release all hook-wires [20G (0.9 mm) 10 cm non-repositionable breast localization needles]. Finally, the trocar was removed after the release. CT scans were performed again to assess the positional relationship between the localization material and the pulmonary nodules, as well as to determine any complication occurrence. After the positioning procedure, the patient was transferred to the operating room or ward to await surgery.

### 2.4 VATS

**Figure 1:**
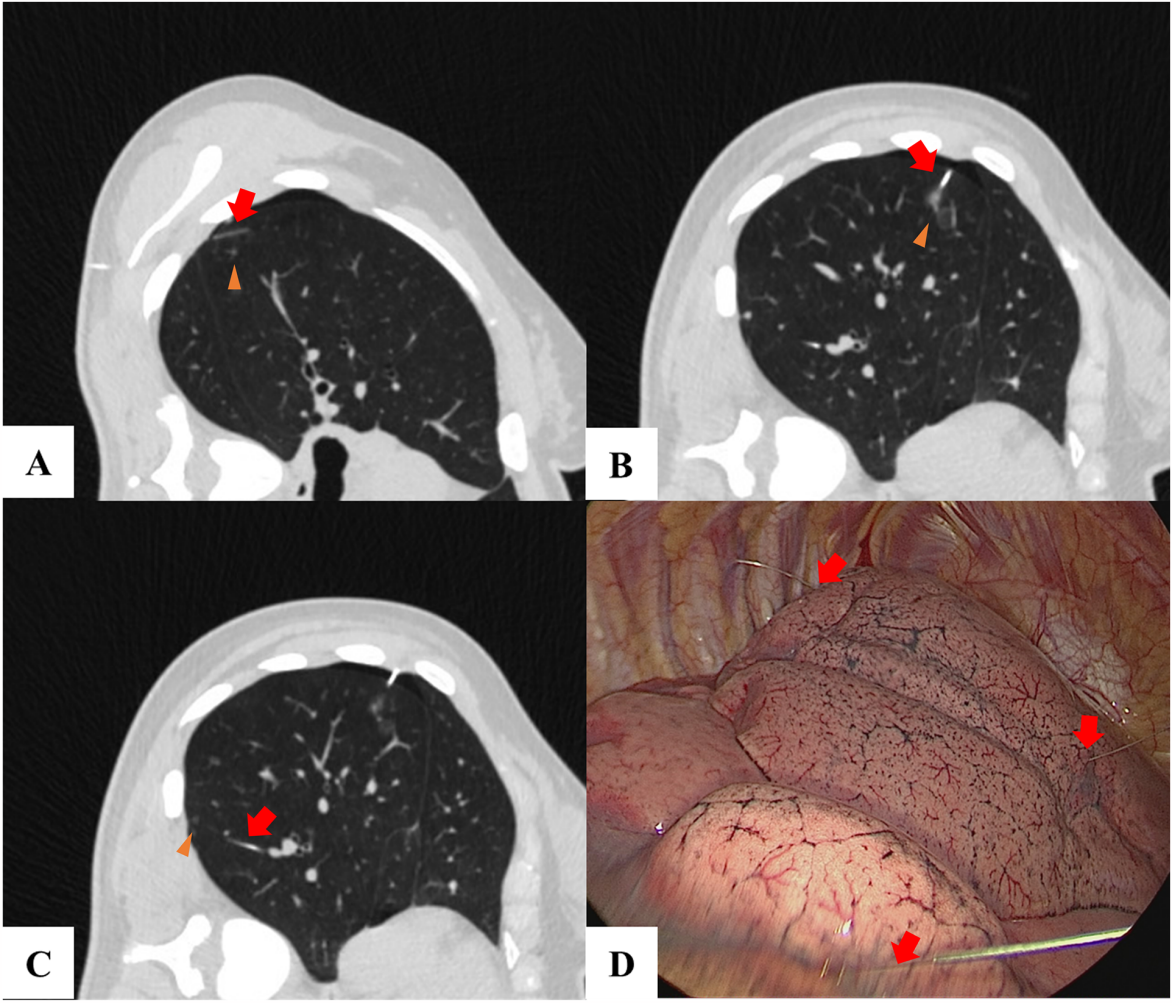
A patient with 3 lung nodules used hook-wire for localization. **(A–C)** A lung nodule (triangles) was located and the hook-wire (red arrow) was inserted near the nodule. **(D)** The hook-wire (red arrow) was visualized during the VATS.

**Figure 2:**
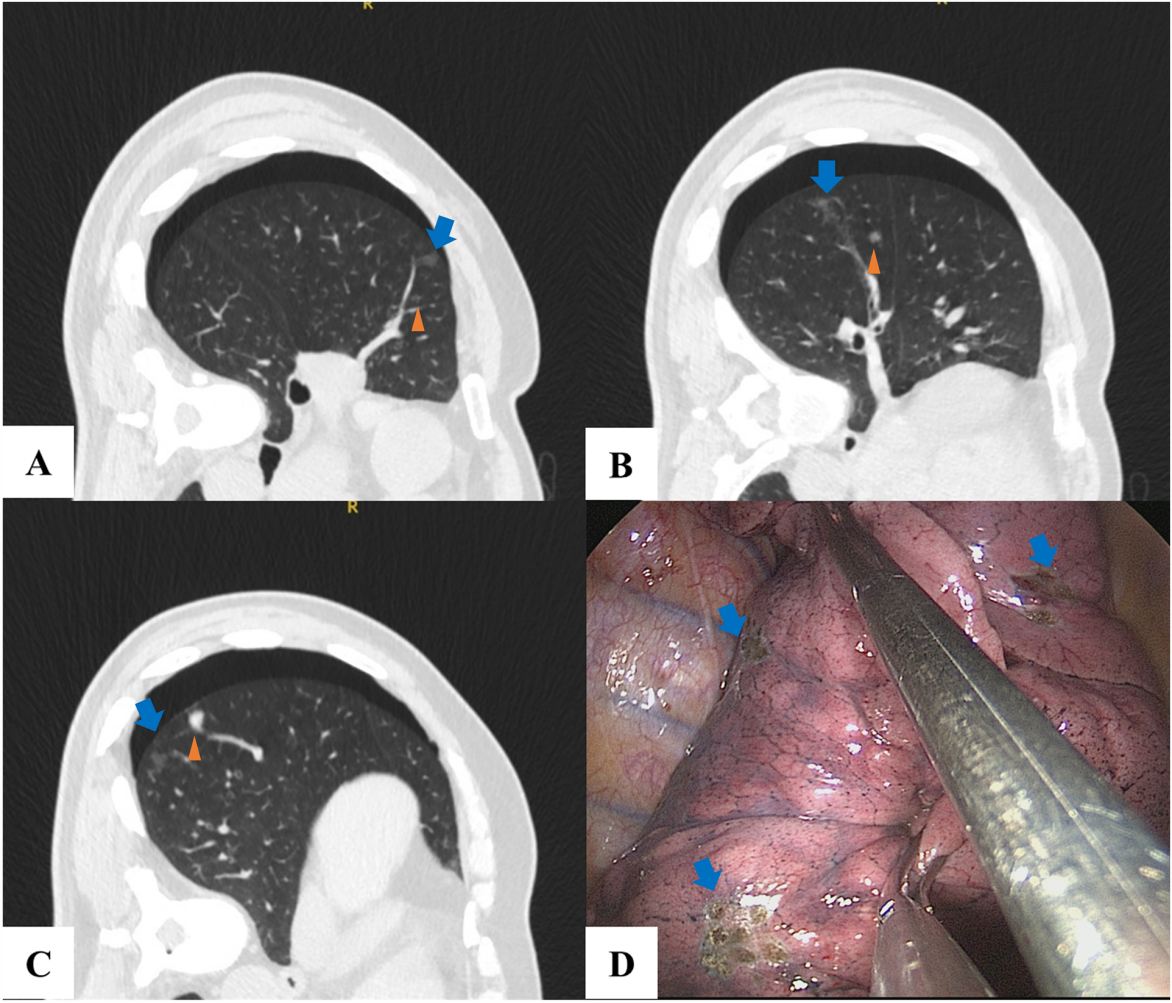
A patient with 3 lung nodules used medical glue for localization. **(A–C)** A lung nodule (triangles) was located and the medical glue (blue arrow) was injected near the nodule. **(D)** During VATS, the pulmonary nodule was identified, and electrocautery was applied to the pleura to delineate its approximate location (blue arrow).

The VATS procedure was performed based on the guidance of the localization CT images (Figures 1, 2), thus ensuring that the localization material was accurately placed and the pulmonary nodules were completely resected.

At our institution, we adhere to a rigorous intraoperative protocol to ensure complete resection of pulmonary nodules. Following tissue resection, systematic visual inspection of the dissected lung parenchyma is performed to identify the target nodule and grossly evaluate resection margins. To objectively confirm margin status, all resected pulmonary nodules are routinely submitted for frozen section examination without exception. For lobectomy specimens containing multiple nodules, each individual lesion undergoes separate pathological evaluation to verify complete excision.

### 2.5 Statistical analysis

The medical glue group represented the experimental group, while the hook-wire group constituted the control group. The primary study endpoint was the localization success rate. According to earlier studies, the success rate of medical glue for localizing multiple pulmonary nodules was 98% (7), whereas that of hook-wire was 90% (6). The sample size ratio between the experimental and control groups was set at 1:1, with a two-sided *α* of 0.05 and a power of 1−*β* = 0.9. Using R software, the required sample sizes for the experimental and control groups were calculated to be 36 patients for both groups. Considering a 10% loss to follow-up and refusal rate, at least 40 patients were required in each group. Thus, the final total sample size was 80 patients. Furthermore, a non-inferiority margin of 10% was determined to be appropriate based on clinical practice findings. Accordingly, non-inferiority was considered significant if the upper limit of the 95% confidence interval (CI) for the absolute difference in success rates was <10%.

Descriptive statistics for continuous variables were expressed as means ± standard deviation (SD). Continuous variables were analyzed using Student's *t*-test, while categorical variables were compared by conducting *χ*^2^ or Fisher's exact tests. Additionally, repeated measures data were assessed utilizing repeated measures analysis of variance. All statistical analyses were performed using SPSS software (version 25.0, IBM). *P* < 0.05 was considered statistically significant. Binary logistic regression analysis was employed to identify risk factors associated with pain, with the results presented as odds ratios and corresponding 95% CIs. Statistically significant variables with *P* < 0.05 in univariate analysis were included in multivariate analysis.

## 3 Results

### 3.1 Characteristics of patients and nodules

**Figure 3:**
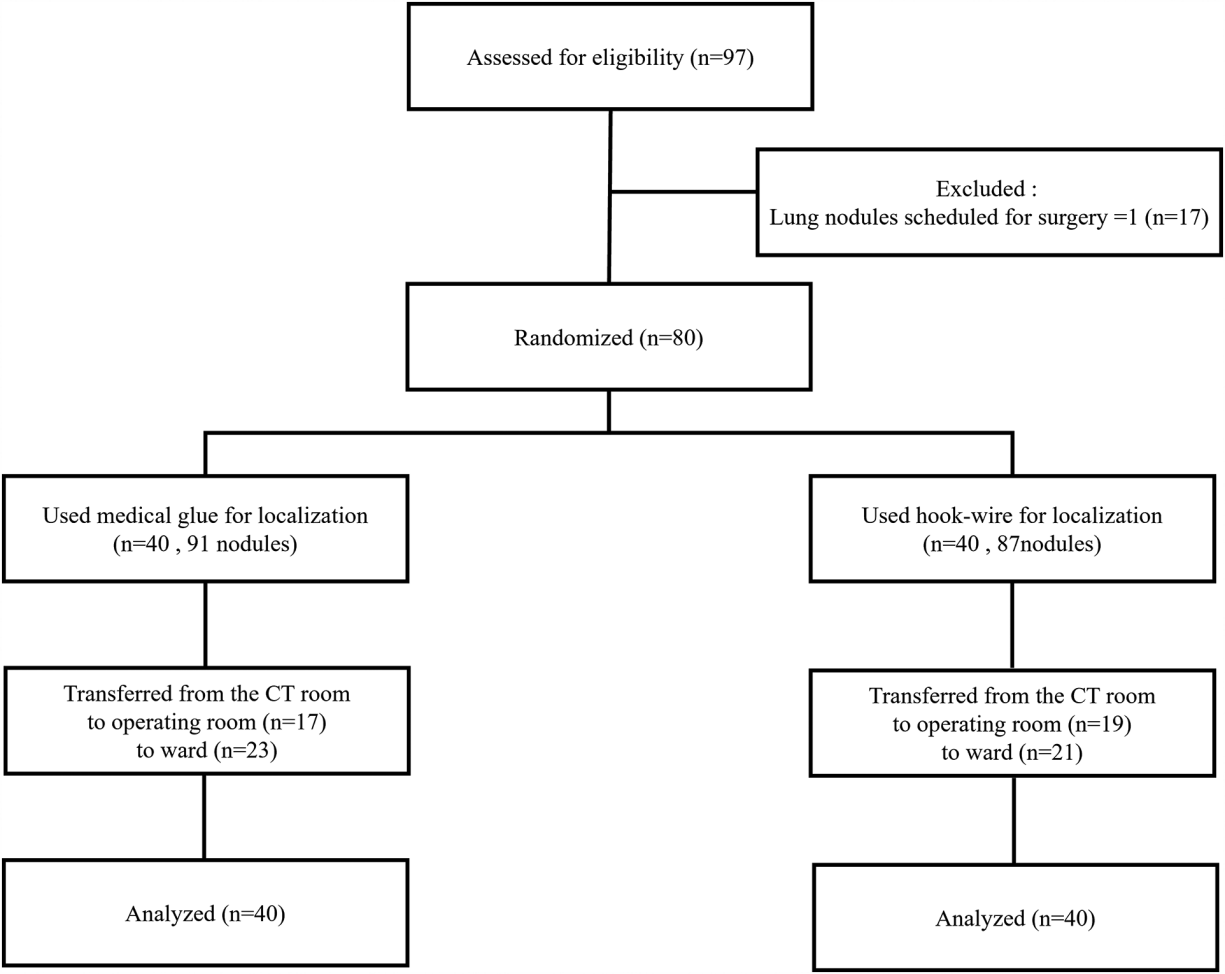
Flow chart of patient selection.

A total of 80 patients with 178 pulmonary nodules were included in this study. Under CT guidance, 40 patients with 91 lesions underwent localization using medical glue, including 29 with two nodules and 11 with three nodules. Additionally, 40 patients with 87 lesions received hook-wire localization, among which 33 had two nodules and seven had three nodules. No significant differences in baseline patient data and nodule characteristics were observed between the two groups (all *P* > 0.05) (Table 1). A flowchart of the selection process of the study patients is shown in Figure 3.

**Table 1:** Characteristics of patients and nodules.

Variables	Medical glue group (*n* = 40)	Hook-wire group (*n* = 40)	*P* value
Age (years)	50.88 ± 13.40	54.27 ± 11.36	0.225
BMI	24.08 ± 2.27	23.54 ± 3.08	0.381
FEV_1_/FVC (%)	82.45 ± 3.93	81.08 ± 4.15	0.132
Sex			0.626
Male	11 (27.50)	13 (32.50)	
Female	29 (72.50)	27 (67.50)	
Hypertension	5 (12.50)	9 (22.50)	0.239
Diabetes	1 (2.50)	6 (15.00)	0.113
CAD	4 (10.00)	3 (7.50)	1.000
Emphysema	1 (2.50)	4 (10.00)	0.356
Number of lesions			0.284
2	29 (72.50)	33 (82.50)	
3	11 (27.50)	7 (17.50)	
Localization laterality			0.485
Right	13 (32.50)	16 (40.00)	
Left	27 (67.50)	24 (60.00)	
Same lobe			0.340
No	29 (72.50)	25 (62.50)	
Yes	11 (27.50)	15 (37.50)	
Distance from pleural surface to the nodule (cm)	1.09 ± 0.97	1.07 ± 0.96	0.879
CT nodule size (cm)	0.80 ± 0.46	0.79 ± 0.44	0.952
Nodule location			0.833
RUL	24 (26.37)	19 (21.84)	
RML, RLL	36 (39.56)	33 (37.93)	
LUL	13 (14.29)	15 (17.24)	
LLL	18 (19.78)	20 (22.99)	
Nodule image characteristics			0.541
Pure GGO	58 (63.74)	51 (58.62)	
Part-solid GGO	14 (15.38)	19 (21.84)	
Solid	19 (20.88)	17 (19.54)	

BMI, body mass index; FEV_1_, forced expiratory volume in 1 s; FVC, forced vital capacity; CAD, coronary artery disease; CT, computed tomography; LUL, left upper lobe; LLL, left lower lobe; RUL, right upper lobe; RML, right middle lobe; RLL, right lower lobe. GGO, ground glass opacity.

### 3.2 Localization outcomes

The localization-related data are presented in Table 2. The localization success rate of the medical glue group was 97.5%, with one failure occurring in a patient requiring two localization sites. Furthermore, intraoperative exploration revealed that one of the medical glue applications was on the parietal pleura and did not penetrate the lung tissue. In the case of the hook-wire group, the localization success rate was 95%, with failure experienced in two patients. In one patient who required two localization sites, the needle tip at the lower lobe site was inserted exceedingly superficially and failed to penetrate the pleura, resulting in a failed localization. In the other patient, CT scans indicated successful localization; however, intraoperative exploration revealed that one of the three hook-wires had been dislodged. Accordingly, the absolute difference in success rates between the two groups was 2.5%, with the 95% CI ranging from −5.82% to 10.82%. Given that the lower limit of the 95% CI for the difference in success rates between the medical glue and hook-wire groups was greater than the non-inferiority margin of −10%, the success rate of medical glue for the CT-guided localization of multiple pulmonary nodules was not inferior to that of hook-wire.

**Table 2:** Localization outcomes.

Variables	Medical glue group (*n* = 40)	Hook-wire group (*n* = 40)	*P* value
Successful localization	39 (97.50)	38 (95.00)	1.000
Number of CT scans	4.28 ± 1.75	4.20 ± 1.70	0.836
Procedure duration (min)	15.68 ± 3.96	15.62 ± 4.35	0.846
Position change	5 (12.50)	5 (12.50)	1.000
Distance from the localization material to the nodule (cm) (*n* = 88,80)	1.02 ± 0.43	0.85 ± 0.55	**0** **.** **028**
Complications	17 (42.50)	22 (55.00)	0.263
Pneumothorax	9 (22.50)	19 (47.50)	**0** **.** **019**
Hemorrhage	3 (7.50)	11 (27.50)	**0** **.** **019**
Irritant cough	9 (22.50)	0 (0.00)	**0** **.** **005**
Pain scores at 15 min post-procedure	4.50 ± 1.15	4.00 ± 1.43	0.090
Localization-to-surgery interval duration (h)	3.43 ± 2.67	3.19 ± 2.50	0.686
Time interval between localization and operation ≥ 2 h	23 (57.50)	21 (52.50)	0.653
Morphine use (*n* = 23,21)	2 (8.70)	4 (19.05)	0.576
Pain scores at 2 h post-procedure(*n* = 23,21)	2.70 ± 0.76	3.71 ± 1.42	0.007

*P* values < 0.05 are in boldface.

Additionally, no significant differences were found in terms of localization duration, number of CT scans, position change rates, and overall complication rates between the medical glue and hook-wire groups. However, the incidence of pneumothorax and pulmonary hemorrhage in the medical glue group was significantly lower than that in the hook-wire group (pneumothorax: 22.5% vs. 47.5%, *P* = 0.019; pulmonary hemorrhage: 7.5% vs. 27.5%, *P* = 0.019). The medical glue group also demonstrated an incidence rate of 22.5% (9/40) for the complication of irritant cough. The distance between the localization material and the target nodule was measured on preoperative CT images in patients with successful localization. The analysis revealed a significantly greater distance in the medical glue group compared to the hook-wire group (1.02 ± 0.43 cm vs. 0.85 ± 0.55 cm, *P* = 0.028).

Moreover, no significant differences were noted between the two groups in terms of pain scores 15 min after localization, duration of the localization-to-surgery interval, or the rate of morphine use during the localization-to-surgery interval. A total of 44 patients had a localization-to-surgery interval of >2 h, with 23 in the medical glue group and 21 in the hook-wire group. However, the pain scores 2 h after localization were significantly lower in the medical glue than in the hook-wire group (2.70 ± 0.76 vs. 3.71 ± 1.42, *P* = 0.007).

### 3.3 Surgical outcomes

VATS for lung nodule resection had a 100% success rate in the medical glue and hook-wire groups, with no patients requiring an extended resection. Nodules that could not be localized utilizing preoperative CT-guided techniques were successfully localized using the anatomical landmark method during surgery, and all targeted nodules were successfully resected.

No significant differences in the type of surgical resection, surgery duration, pathological types, or postoperative hospital stay were detected between the two groups (all *P* > 0.05) (Table 3).

**Table 3:** Surgical outcomes.

Variables	Medical glue group (*n* = 40)	Hook-wire group (*n* = 40)	*P* value
Duration of VATS (h)	2.20 ± 1.18	1.87 ± 0.78	0.145
Postoperative hospital stay (day)	4.82 ± 2.70	5.00 ± 1.74	0.724
Pathologic diagnosis			0.472
Benign	12 (13.19)	14 (16.09)	
Atypical adenomatous hyperplasia	6 (6.59)	4 (4.60)	
Adenocarcinoma *in situ*	13 (14.29)	10 (11.49)	
Minimally invasive adenocarcinoma	45 (49.45)	36 (41.38)	
Invasive adenocarcinoma	15 (16.48)	23 (26.44)	
Type of resection			0.366
Wedge resection	6 (15.00)	11 (27.50)	
Wedge resection + Wedge resection	31 (77.50)	25 (62.50)	
Wedge resection + Lobectomy	2 (5.00)	1 (2.50)	
Lobectomy	1 (2.50)	3 (7.50)	

### 3.4 Risk factors for post-localization pain

Patients who required morphine analgesia during the localization-to-surgery interval and those with a pain score of >3 at 2 h after localization were categorized into a moderate-to-severe pain group, whereas the remaining patients were allocated to a mild pain group. A binary logistic regression analysis revealed that factors such as age, gender, BMI, lung function, and lesion location were not significantly associated with post-localization pain (all *P* > 0.05). However, the duration of the localization procedure and the type of localization material employed were identified as independent risk factors for pain. Further multivariate analysis of the duration of the localization procedure and the type of localization material revealed that localization material was an independent influencing factor of moderate-to-severe pain after localization (Table 4).

**Table 4:** Risk factors for post-localization pain.

Variables	Univariable			Multivariable		
	OR	95% CI	*P*	OR	95% CI	*P*
Sex						
Male	1.00					
Female	0.45	0.12–1.76	0.255			
Age	1.02	0.96–1.08	0.485			
BMI	0.95	0.75–1.19	0.637			
FEV_1_/FVC (%)	1.00	0.85–1.18	0.988			
Procedure duration	0.72	0.54–0.97	**0** **.** **030**	0.76	0.56–1.03	0.073
Nodule laterality						
Left	1.00					
Right	0.39	0.10–1.54	0.179			
Same lobe						
No	1.00					
Yes	0.56	0.13–2.46	0.439			
Position change						
No	1.00					
Yes	1.80	0.36–9.08	0.476			
Materials						
Hook-wire	1.00			1.00		
Medical glue	0.10	0.02–0.56	**0** **.** **009**	0.12	0.02–0.72	**0** **.** **020**

OR, odds ratio; CI, confidence interval.

*P* values <0.05 are in boldface.

## 4 Discussion

The accurate and safe preoperative localization of lung nodules is a prominent challenge for surgeons performing VATS (11). Currently, the CT-guided localization of lung nodules is the most widely utilized method, with hook-wire being the most commonly employed localization material. However, hook-wire application for multiple lung nodules can lead to higher rates of chest pain and complications (7). Therefore, selecting the appropriate localization material is crucial in patients with multiple pulmonary nodules. In this study, we compared the effectiveness and safety of medical glue and hook-wire for the CT-guided localization of multiple lung nodules. Our findings revealed that the success rate of medical glue-guided localization was not inferior to that of hook-wire-guided localization. Furthermore, medical glue localization demonstrated advantages over hook-wire localization in reducing post-procedural complications and pain.

In this study, successful localization was defined as achieving a distance of <2 cm between the localization material and the target nodule, along with intraoperative exploration results confirming that the localization material was not displaced. Based on this definition, our results showed that the medical glue and hook-wire groups had localization success rates of 97.5% and 95%, respectively. This outcome indicates that the success rate of medical glue was not inferior to hook-wire, underscoring that both materials are effective for localization. However, hook-wire has been shown to have poor stability. For example, the muscular activity of patients following the localization procedure can lead to wire displacement or slippage, which causes localization deviation and ultimately affects surgical accuracy and success (12). Previous studies have reported a hook-wire displacement rate ranging from 2.7% to 7.3% (12–14). In this study, localization had failed in one patient in the hook-wire group due to displacement. In contrast, medical glue solidifies and does not migrate after being injected into the lung, thus avoiding issues similar to hook-wire displacement. Although the localization success rate of medical glue was slightly higher than that of hook-wire in this study, the obtained data were inadequate to confirm its superiority.

The withdrawal of the trocar from the lung tissue during the localization procedure can cause air to be released from the puncture site at the visceral pleura, leading to pneumothorax. The pleura collapses following pneumothorax, hindering the ability to provide the necessary tension to support the trocar passage through the pleura and thus complicating the localization procedure. In the case of multiple lung nodules, pneumothorax occurrence after the first puncture can make it challenging to perform additional punctures for the remaining nodules (10). In this study, pneumothorax incidence was significantly lower in the medical glue group than in the hook-wire group, underlining the advantage provided by medical glue in localizing multiple lung nodules. Moreover, pneumothorax incidence has been found to vary with the different types of puncture trocar. For example, finer needles cause less damage to the pleura and lung tissue, thereby lowering pneumothorax risk (11). Hook-wire localization uses a 20G trocar, while medical glue localization utilizes a thinner 22G trocar. Additionally, medical glue possesses adhesive properties that can effectively prevent the extension of the pneumothorax localization-to-surgery interval (7).

The current study also showed a significantly lower incidence of lung hemorrhage in the medical glue group than in the hook-wire group. In hook-wire localization, the distal end is fixed to the chest wall, while the wire within the lung can shift according to the patient's breathing. This feature can increase the risk of damage to small pulmonary vessels and cause bleeding incidence. In contrast, the coagulation process of the medical glue aids in sealing damaged blood vessels, thereby reducing bleeding risk. However, the glue releases gas during the coagulation process, which may induce irritant coughing in patients. This finding aligns with our study results, where nine patients in the medical glue group experienced coughing, while none in the hook-wire group reported this symptom. Prior studies have suggested that the rapid release of glue (>0.05 ml/s) and the excessive depth of injection are factors contributing to this complication (9). Furthermore, no air embolism or other severe complications were observed in this study. Although severe complications are extremely rare, an air embolism occurrence rate of 0.5% has been demonstrated after hook-wire localization (15). Conversely, no incidence of air or large vessel embolism has been observed during medical glue application for preoperative localization.

Binary logistic regression analysis revealed that the duration of the localization procedure and the type of localization material were independent risk factors for pain after localization. In multivariable analysis, the type of localization material exhibited a more significant effect on pain than procedure duration. This result suggests that patient pain following localization can be reduced by prioritizing the selection of the appropriate material in clinical practice. Earlier studies have demonstrated conflicting results in terms of whether medical glue localization leads to more severe pain than hook-wire localization. For instance, Wang H et al. found that a higher proportion of patients in the medical glue group experienced severe pain than the hook-wire group (6.10% vs. 3.68%) (16). In contrast, research by Huang Y showed that chest pain incidence was lower in the medical glue group than in the hook-wire group (3% vs. 16%) (9). However, these studies did not specify the time points for measuring pain. In our study, pain scores were recorded 15 min and 2 h after localization. The present study results indicated that the medical glue group had a higher pain score than the hook-wire group at 15 min following localization, although this difference was not statistically significant. In contrast, the pain score at 2 h was significantly lower in the medical glue group than in the hook-wire group. The coagulation process of medical glue during localization releases heat, which can reach a peak temperature of up to 64 °C within 30 s. This released heat may irritate the pleura and cause intense chest pain immediately after localization (17). However, this irritation to the pleura substantially decreases after the glue completely solidifies (10). In the case of hook-wire localization, the distal end of the wire remains fixed to the chest wall. Consequently, irritation to the intercostal nerves and pleura persists, resulting in continuous discomfort for the patient. These findings explain the significant decrease in the pain score in the medical glue group compared to that in the hook-wire group at 2 h after the localization procedure.

The application of medical glue requires a higher level of technical skill from operators compared to hook-wire localization. In our center, the depth of the medical glue cannula needle is set at 0.5 cm. If the medical glue is injected too deeply, the resulting coagulum may become difficult to palpate during VATS, potentially hindering effective intraoperative guidance (Figure 2). Additionally, the trocar must be positioned slightly distal to the target nodule. This is important because the diffusion of medical glue into the surrounding alveolar tissue during coagulation may compromise pathological sampling if the needle is placed too close to the target (9). These technical considerations can lead to a greater distance between the medical glue and the target nodule, which may be especially significant when localizing deeper nodules.

This study has several limitations that should be considered. One of the shortcomings was that this study was a single-center, small-scale investigation with a limited sample size. Hence, larger, multicenter, prospective studies with larger sample sizes are needed to further validate our findings. Another limitation of this study was that it only involved the localization of two to three nodules. Consequently, we did not perform subgroup comparisons based on the number of localized nodules. However, a more detailed subgroup analysis is required to compare the surgical outcomes of different numbers of localized nodules. All these limitations highlight the need for additional research to confirm our results and explore the effects of varying clinical conditions.

## 5 Conclusion

The application of medical glue for the CT-guided localization of multiple pulmonary nodules demonstrates a success rate comparable to that acquired through hook-wire localization. Compared to hook-wire, medical glue is associated with a lower incidence of complications such as pneumothorax and lung hemorrhage. Additionally, the medical glue group showed lower pain scores than the hook-wire group at 2 h after localization. All these findings suggest that medical glue is a safe and effective alternative to hook-wire for localizing multiple lung nodules, leading to fewer complications and reduced pain.

## Data Availability

The raw data supporting the conclusions of this article will be made available by the authors, without undue reservation.
